# Effects of vitamin D3 supplementation on serum 25(OH)D concentration and strength in athletes: a systematic review and meta-analysis of randomized controlled trials

**DOI:** 10.1186/s12970-019-0323-6

**Published:** 2019-11-26

**Authors:** Qi Han, Xueyang Li, Qiushi Tan, Jing Shao, Muqing Yi

**Affiliations:** 1 National Research Institute of Sports Medicine, Beijing, China; 2 Beijing Sport University, Beijing, China; 3 Institute of Scientific and Technical Information of China, Beijing, China

**Keywords:** 25(OH)D, Exercise, Muscle, Strength and conditioning, Physical fitness

## Abstract

**Background:**

The purpose of this systematic review and meta-analysis is to investigate the effects of vitamin D3 supplementation on skeletal muscle strength in athletes. Vitamin D3 supplements or vitamin D3 fortified foods always have claims for bringing people health benefits including bone and muscle health. An up-to-date rigorous systematic review and meta-analysis is important to better understand the effect of vitamin D3 supplementation on muscle strength.

**Methods:**

English written randomized controlled trials (RCTs) that looked at effects of vitamin D3 supplementation on muscle strength in healthy athletes were searched using three databases (PubMed, Embase and Cochrane Library). Serum 25(OH)D above 30 ng/mL is considered to be sufficient in this systematic review and meta-analysis.

**Results:**

Five RCTs with 163 athletes (vitamin D3 *n* = 86, placebo *n* = 77) met inclusion criteria. Fourteen athletes were lost to follow-up and 149 athletes (vitamin D3 *n* = 80, placebo *n* = 69) were documented with complete result. Among athletes with baseline serum 25(OH)D values suggesting insufficiency, vitamin D3 daily dosage at 5000 IU for over 4 weeks led to a serum 25(OH)D concentration of 31.7 ng/mL. Athletes with sufficient serum 25(OH)D level at baseline were recruited in only one study, and the participants of which were assigned to either vitamin D3 at a daily dosage of 3570 IU or placebo for 12 weeks, their serum 25(OH)D sufficiency (VD: 37.2 ± 7.6 vs. 45.6 ± 7.6; PL: 38 ± 6.8 vs. 32 ± 8.4) was well maintained above the cut-off boundary. One repetition maximum Bench Press (1-RM BP) was not improved significantly (SMD 0.07, 95% CI: − 0.32 to 0.47, *P* = 0.72) and there was no significant increase in maximal quadriceps contraction (SMD -2.14, 95% CI: − 4.87 to 0.59, *P* = 0.12). Furthermore, there was no significant overall effect of vitamin D3 intervention on muscle strength in this meta-analysis (SMD -0.75, 95% CI: − 1.82 to 0.32, *P* = 0.17).

**Conclusion:**

Although, serum 25(OH)D concentrations after supplementation reached sufficiency was observed, muscle strength did not significantly improve at this point of current meta-analysis. Additional well-designed RCTs with large number of participants examined for the effect of vitamin D3 supplementation on serum 25(OH)D concentrations, muscle strength in a variety of sports, latitudes and diverse multicultural populations are needed.

## Background

Vitamin D is a group of vitamins, which contribute to healthy body function [1]. Without vitamin D, our body cannot absorb calcium, which is a primary component of the bone [2]. In the past century, vitamin D deficiency is heavily studied and reported that vitamin D deficiency is related to several health problems, such as osteoporosis [1–3], muscle aches and weakness [4]. Vitamin D research is becoming an important chapter in sports science and it is reported having beneficial effect on physical fitness, healthy bone structure and skeletal muscle health [5, 6].

Vitamin D2 and D3 acquired from food, sunlight exposure or supplementation can all be converted to 25-hydroxyvitamin [25(OH)D] in the liver and then measured in blood [7]. Then, 25(OH)D can be converted to the bioactive compound calcitriol [1,25(OH)_2_D] in kidney [8]. 1,25-dihydroxyvitamin D3 [1,25(OH)_2_D3] stimulates intestinal absorption of calcium and phosphate and promotes new bone formation [8]. In vivo, rats lacking the vitamin D receptor (VDR) had down regulated bone health and muscle function [9].

In this study, we adopted that serum 25(OH)D concentration above 30 ng/mL shall be considered sufficient as recommended [10, 11]. Maintaining good status of serum 25(OH)D seems bring beneficial impact on athletic performance [12]. However, people are worried that serum 25(OH)D can be double edged sword when it is above 100 ng/mL introducing its toxicity [13]. Its underlying beneficial mechanism for enhancing athletic performance is still under debate. Elevated 1,25(OH)_2_D status and the expression of VDR in muscle cells could play a direct role on calcium binding efficiency for muscle fiber twitch [12], meanwhile, its long-run mechanism could be 1,25-dihydroxyvitamin D increases the size and number of fast twitch muscle fibers [14, 15] and accelerates lipolysis [16] in the TCA cycle.

It was reported that athletes have high prevalence vitamin D insufficiency, which is because they have higher metabolic rate, experiencing all year round indoor training, lacking the sunlight ultraviolet exposure, not having adequate solutions for monitoring and maintaining serum 25(OH)D from extensive physical activities [17–20]. Indeed, coach, athletes, athletic trainers and sports related health-care professions are concerned about athletes’ serum vitamin D sufficiency and how it associates with strength and conditioning as well as athletic performance.

## Rationale and objectives

Previous vitamin D status reporting systematic review that concerned about muscle strength consists of small trials and reported small effect findings [21, 22], and there are reviews focused on effect of vitamin D on muscle function and athletes’ performance [23, 24]; however, we are not finding many up-to-date high quality meta-analysis and systematic review examining the effects of vitamin D3 supplementation among athletes. Here, we proposed to have a systematic review and meta-analysis based on up-to-date high quality randomized controlled trials (RCTs) to improve statistical power. Therefore, in this study, we hypothesized that there is an overall beneficial effect of vitamin D3 supplementation on serum 25(OH)D and muscle strength.

## Methods

### Design

This systematic review and meta-analysis was prepared and conducted in accordance to PRISMA (Preferred Reporting Items for Systematic Reviews and Meta-Analysis) statement [25] to ensure rigorous methodology and reporting.

### Eligibility criteria

The PICO approach was defined as follows: Population (P) was defined as healthy male and female athletes aged 10–45 years old involved in any sport professions. Intervention (I) was oral administration of vitamin D3 supplementation, not limited to any dosage or duration. Comparison (C) was between intervention and placebo. Outcomes (O) were primarily serum 25(OH)D and, secondly, muscle strength.

Only RCTs were included. The eligibility criteria were set to target all trials conducted among athletes. Non-randomized trials, studies without full text, non-athlete trials, vitamin D2 administration, not addressing muscle strength tests, and multivitamin supplementation were excluded. Paralympic athletes and athletes with illness that could influence serum 25(OH)D concentrations or alter their responses to vitamin D3 supplementation were excluded. Research were also excluded if including interventions affecting serum 25(OH)D levels besides vitamin D3 usage, not reporting sufficient information on its quality and having incomplete outcomes.

### Search methods for identification of articles

Literature search of PubMed, Embase, and Cochrane Library databases from inception to May 2019 was accomplished. The following terms and medical subject headings (MeSH) were searched: Vitamin D, supplementation, vitamin D2, vitamin D3, 1-alpha hydroxyvitamin D3, 1-alpha hydroxycalciferol, 1,25-dihydroxyvitamin D3, 1,25 dihydroxycholecalciferol, 25 hydroxycholecalciferol, 25-hydroxyvitamin D, calcitriol, ergocalciferol, cholecalciferol, calcifediol, alfa-calcidol, calcidiol, calciferol, supplementation, supplement, muscle, muscle function, muscle strength, force, power, performance, athletic performance. Duplicates were then removed at the stage of title and abstract assessment with assistant from Mendeley tools and by its notes from Cochrane library.

### Eligibility assessment, study selection, and quality assessment

PRISMA flow diagram and the Cochrane risk of bias (ROB) assessment tool were used to screen, select, and assess the quality of trials. Studies were screened in accordance with PRISMA checklist. Titles and abstracts were reviewed for eligibility by two authors independently. Then, two reviewers independently assessed the full text of these article and their methodological quality, outcomes and duplication. Disagreements were resolved through consensus.

### Data extraction

Data were extracted independently by two authors, disagreements were resolved upon consensus. Athletes’ baseline age, gender, study latitude, sport activities, vitamin D3 (unit, dosages, product, and duration), and outcome measures (mean, SD, unit) were extracted. The dosage of vitamin D3 supplementation varied between trials, and we converted all of them to a daily dosage with international units (IU), where 100 IU is equal to 2.5 μg. Serum 25(OH)D from different trials are reported in ng/mL for consistency, where 1 ng/mL equals to 2.5 nmol/L. SD was extracted from range, standard errors, confidence intervals (CIs) or *p* values if not reported.

### Data stratification and subgroups

During data extraction, we noted that four trials were conducted during wintertime when sunlight exposure is minimal of the year, with only one trial conducted in Fall. Durations of intervention among different studies were approximated at 1, 4, 6, 8 and 12 weeks. Therefore, for consistency, trials were stratified by baseline vitamin D sufficiency for observing vitamin D3 supplementation effects on serum 25(OH)D. And since different muscle strength measurements were applied among included RCTs, we set subgroups of muscle strength outcomes based on muscle strength test.

### Data synthesis

We calculated the baseline pre-supplementation mean difference between vitamin D3 groups and placebo groups. For between-group baseline 25(OH)D status, we performed standardized mean differences (SMDs) check for serum 25(OH)D between vitamin D3 and placebo groups using a random-effects model and an inverse variance approach. Heterogeneity was tested using the Cochran’s Q test with *p* value set at 0.05 for significance and quantified using the I^2^ statistic (I^2^ < 40% as low, 40–60% as moderate, and > 60% as substantial heterogeneity). Review Manager 5.3 software [26] was used for analyses.

## Results

### Eligibility assessment and article selection

**Fig. 1:**
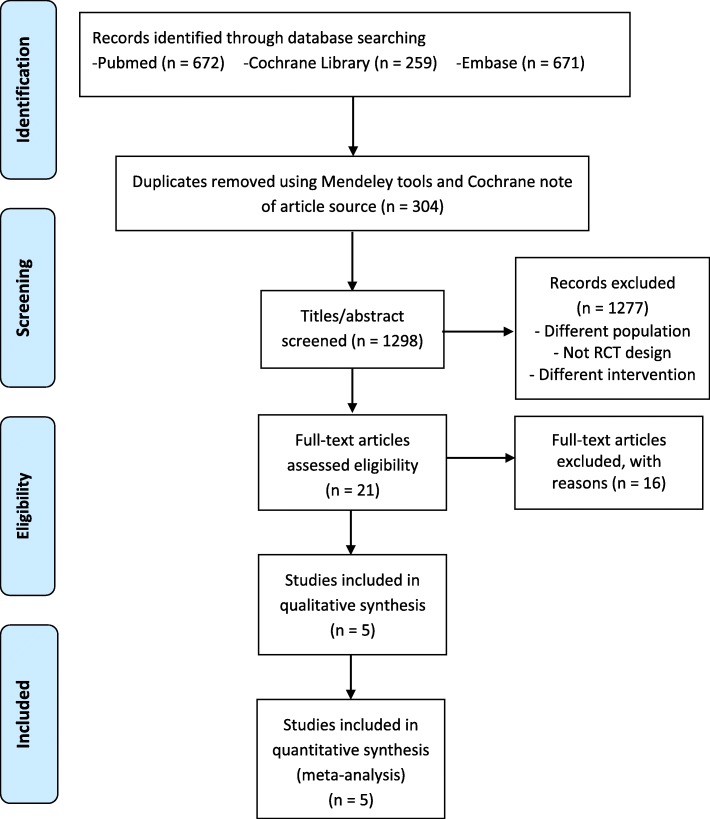
PRISMA flow diagram of search and selection process

Figure 1 summarizes the search and selection process. After reviewing 1298 titles and abstracts, 21 articles were selected for full-text article review. Of the 21 articles, five RCTs were included in this meta-analysis. For example, of the excluded articles, research from Jastrzebska M. et al. [27] was excluded from meta-analysis due to not conducting one repetition maximum Bench Press (1-RM BP) and maximal quadriceps contraction as strength test.

### Publication Bias

**Fig. 2:**
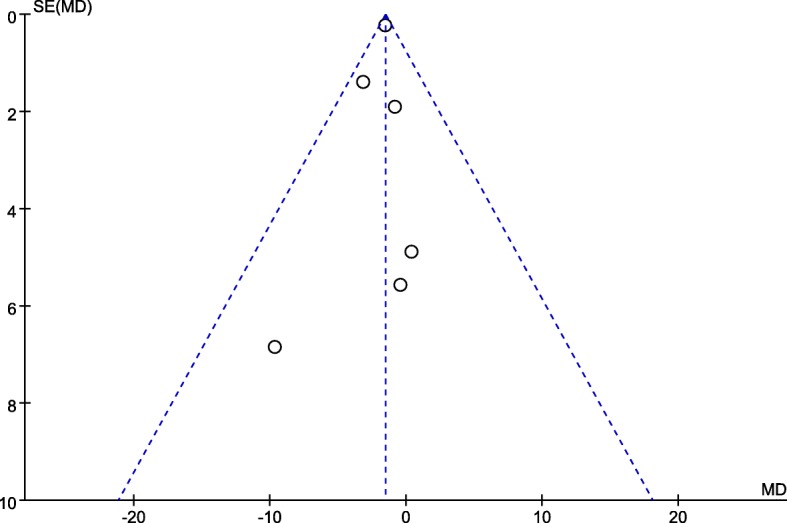
Funnel plot for within study serum 25(OH)D difference between intervention and placebo for each trial at baseline. SE, standard error; MD, mean difference of serum 25(OH)D between intervention and placebo. Close 2013a included 2 different vitamin D3 dosage intervention groups in their study [28]

Figure 2 presents the funnel plot of the included trials for within study mean difference of serum vitamin D status between groups at baseline. The horizontal axis presents within study mean difference of serum 25(OH)D between intervention and placebo for each trial at the baseline [28–32]. The overall heterogeneity test for all selected trials indicating low heterogeneity (I^2^ = 0%, *P* = 0.70), which can be interpreted to have low selection and publication bias for this systematic review and meta-analysis.

### Risk of Bias assessment

**Fig. 3:**
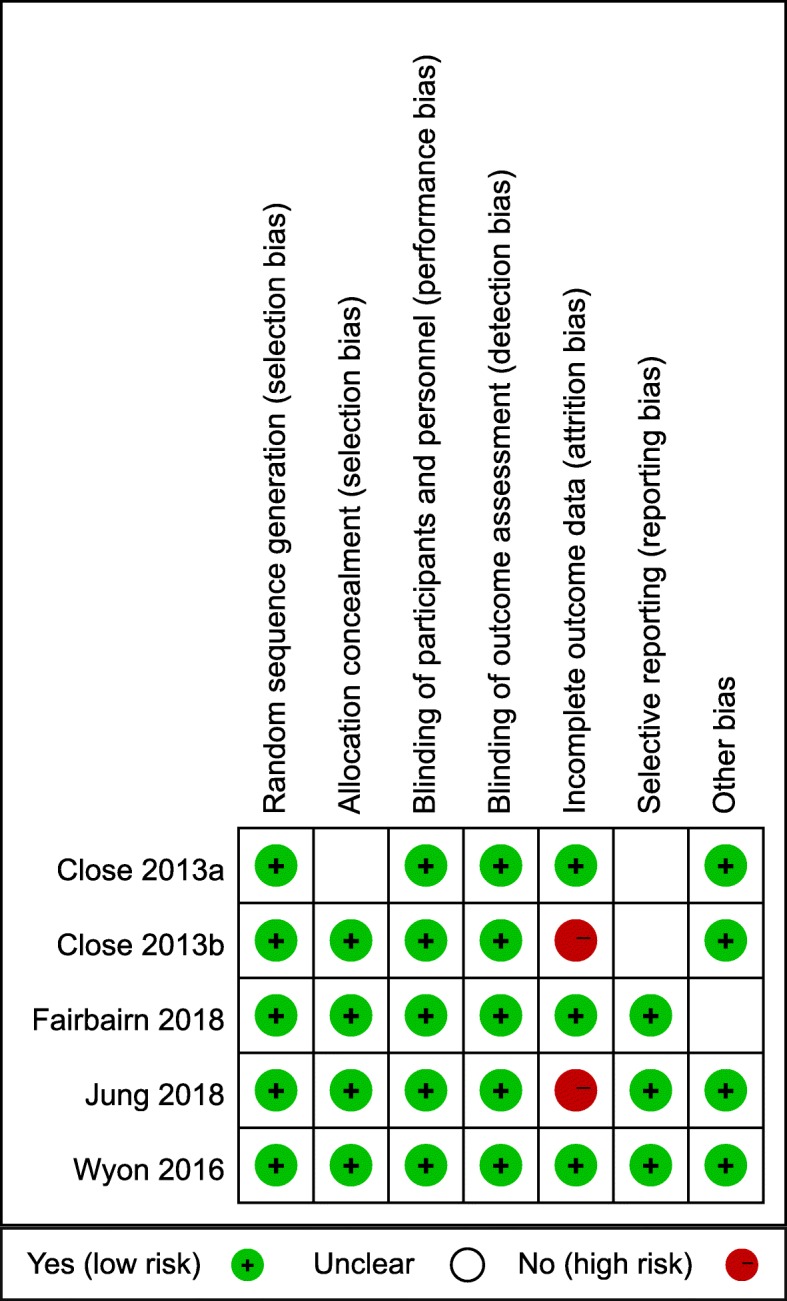
Cochrane risk of bias assessment. 2013a, 30 football and rugby athletes were recruited in reference 28; 2013b, 10 soccer players were recruited in reference 32

Methodological quality of the trials and introduced risk of bias are shown in Fig. 3. Five included studies are all placebo controlled and double blinded studies.

### Trials and participants’ baseline characteristics

Baseline characteristics of subjects from all five included RCTs for analytical and quantitative synthesis are presented in Tables 1 and 2. Three studies were from UK, one from Korea and one from New Zealand. Athletes were engaged in different sports, and four out of the five trials included males only. Their mean age varied from 18 years old in soccer players [32] to 29 years old in judo athletes [29]. The daily dosage was from as low as 2857 IU for 12 weeks [32] to 18,750 IU for 8 days (a bolus of 150,000 IU) [29].

**Table 1:** Characteristics of the included randomized controlled trials

Reference	Country	Latitude	Season	Sports Activity	Randomized (*n* = 163)	Intervention Vitamin D Dosage IU (μg)	Duration	Type	Product
Jung 2018 [30]	Korea	33.3° N	Winter	Taekwondo	22/22	5000	4 weeks	D_3_ capsules	Bio-Tech Pharmacal, Inc. (Arkansas, USA)
Fairbairn 2018 [31]	New Zealand	45–46.5° S	Autumn	Rugby	28/29	3570 IU (89 μg)/day	11–12 weeks	D_3_ tablets	Cal.D.Forte, PSM Healthcare, Auckland, New Zealand
Close 2013b [32]	UK	53° N	Winter	Soccer	5/5	5000(125)/day vs. PL	8 weeks	D_3_ capsules	Biotech Pharmacal Inc., Phoenix, AZ, USA
Close 2013a [28]	UK	53° N	Winter	Football and Rugby	10/10/10	20,000 (500)/week vs. 40,000 (1000)/week vs. PL	12 weeks	D_3_ capsules	Biotech Pharmacal Inc., Phoenix, AZ, USA
Wyon 2016 [29]	UK	52.3° N	Winter	Judo	11/11	150,000 (3750)/one time vs. PL	8 days	D_3_ tablets	Not reported

Data are means unless stated otherwise; *PL* Placebo. 2013a, 30 football and rugby athletes were recruited in reference 28; 2013b, 10 soccer players were recruited in reference [32]

**Table 2:** Baseline measurements of the included randomized controlled trials

Reference	Analyzed (*n* = 149)	VD3 Daily Dosage IU	Males (%)	Mean Age Years	25(OH)D (ng/mL)	25(OH)D (nmol/L)	25(OH)D Method of Analysis	Lost to Follow-up
Jung 2018 [30]	20	5000	60% (21/35)	20.1 ± 0.15	10.9 ± 0.47	27.3 ± 1.18	CLIA analyzer (Liaison XL, Dasorin, Italy)	9
	15	PL			12.36 ± 0.78	30.9 ± 1.95		(20.45%)
Fairbairn 2018 [31]	28	3570	100	21.5 ± 2.8	37.2 ± 7.6	93 ± 19	LC-MS/MS at Canterbury Health Laboratories, Christchurch, New Zealand	0
	29	PL		20.9 ± 2.8	38 ± 6.8	95 ± 17		
Close 2013b [32]	5	5000	100	18 ± 5	11.6 ± 10	29 ± 25	HPLC-MRM (Becton Dickinson, Oxford, UK)	0
	5	PL			21.2 ± 11.6	53 ± 29		
Close 2013a [28]	6	5714	100	21 ± 1	20.4 ± 10.4	51 ± 26	HPLC-MRM (Becton Dickinson, Oxford, UK)	5
	10	2857		22 ± 2	21.2 ± 10.4	53 ± 26		(16.70%)
	9	PL		21 ± 1	20.8 ± 10.8	52 ± 27		
Wyon 2016 [29]	11	18,750	100	29 ± 10.6	13.2 ± 3.8	32.8 ± 9.4	ECLIA (Tecan Infinite F500, Mannedorf, Switzerland)	0
	11	PL		26 ± 7.4	16.3 ± 2.7	40.7 ± 6.8		

Data are mean ± standard deviation unless stated otherwise. *CLIA* Chemiluminescent immunoassay, *ECLIA* Electrochemiluminescent immunoassay, *HPLC-MRM* High-performance liquid chromatography tandem multiple reaction mode, *HPLC-MS* High-performance liquid chromatography-tandem mass spectrometry, *LC–MS/MS* Liquid chromatography mass spectrometry, *PL* Placebo. 2013a, 30 football and rugby athletes were recruited in reference 28; 2013b, 10 soccer players were recruited in reference [32]

Wyon et al. [29] recruited male Judo athletes in the UK, where they performed nutritional intervention in wintertime with a dosage of one time administration of 150,000 IU vitamin D3 tablets, and the duration between their post-intervention and pre-intervention screening and evaluation was 8 days. Close et al. [28] reported that they had both UK Football and Rugby male athletes assigned to three groups in one study for vitamin D3 nutritional aid capsules for 12 weeks at dosage of 20,000 per week, 40,000 per week or placebo. In another study, Close et al. [32] reported that they had UK male soccer players assigned a daily dosage of 5000 IU vitamin D3 capsules for 8 weeks. For both studies carried out by Close et al. [28, 32], they had vitamin D3 capsules from Biotech Pharmacal Inc., Phoenix, AZ, USA. Jung et al. [30] conducted their study in Korea during winter for both male and female Taekwondo athletes for 4 weeks with a daily dosage of 5000 IU vitamin D3 capsules provided by Bio-tech Pharmacal Inc., Arkansas, USA. Fairbairn et al. [31] had New Zealand Rugby male athletes taken 3570 IU vitamin D3 tablets (Cal.D.Forte, PSM Healthcare, Auckland, New Zealand) daily for 11–12 weeks in the autumn.

### Serum 25(OH)D concentrations

#### Vitamin D3 supplementation effects on serum 25(OH)D status for included research

**Fig. 4:**
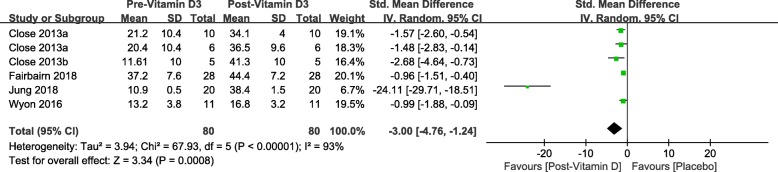
Forest plot for vitamin D3 supplementation effects on serum 25(OH)D status

Both Tables 3 and 4 demonstrate mean serum 25(OH)D concentration at the baseline and follow-up for each study. For those athletes with insufficient serum 25(OH)D at the baseline, vitamin D3 supplementation improved their vitamin D status. Fairbairn [31] reported that athletes with sufficient vitamin D status showed an increase in serum 25(OH)D at a daily dosage of 3570 IU. (Table 3) Fig. 4 is the forest plot for vitamin D3 supplementation effects on serum 25(OH)D status. Wyon et al. [29] assigned their participants with a single bolus of 150,000 IU vitamin D3 supplementation, even though the mean serum 25(OH)D was under 30 ng/mL at the day eight after the dosage, an improved serum 25(OH)D status was observed.

**Table 3:** Baseline and Follow-up Serum 25(OH)D concentrations

Reference	Latitude	Time	Vitamin D3 Daily Dosage	Baseline (ng/mL)	*N* = 149	1 Week (ng/mL)	4 Weeks (ng/mL)	6 Weeks (ng/mL)	8 Weeks (ng/mL)	12 Weeks (ng/mL)
			IU							
Jung 2018 [30]	33.3° N	Jan–Feb	5000	10.9 ± 0.5	20		38.4 ± 1.5			
			0	12.4 ± 0.8	15		13.1 ± 1.0			
Fairbairn 2018 [31]	45–46.5° S	Mar–May	3570	37.2 ± 7.6	28			44.4 ± 7.2		45.6 ± 7.6
			0	38 ± 6.8	29			34 ± 6.8		32 ± 8.4
Close 2013b [32]	53° N	Nov–Jan	5000	11.6 ± 10.0	5				41.3 ± 10.0	
			0	21.2 ± 11.6	5				29.6 ± 9.6	
Close 2013a [28]	53° N	Jan–Apr	5714	20.4 ± 10.4	6			39.3 ± 5.6		36.5 ± 9.6
			2857	21.2 ± 10.4	10			31.7 ± 5.6		34.1 ± 4.0
			0	20.8 ± 10.8	9			14.8 ± 7.2		16.4 ± 8.8
Wyon 2016 [29]	52.3° N	Feb	18,750	13.2 ± 3.8	11	16.8 ± 3.2				
			0	16.3 ± 2.7	11	16.3 ± 2.6				

Data are mean ± standard deviation unless stated otherwise. Measurements are in ng/mL

**Table 4:** Baseline and end-point mean 25(OH)D concentrations in vitamin D and placebo

Reference	Latitude	Vitamin D Daily Dosage (IU)	Weeks	Vitamin D Supplementation^a^				Placebo^a^			
				N	Pre	Post	Change	N	Pre	Post	Change
Insufficient vitamin D (*N* = 92)											
Winter (*N* = 92)											
Jung 2018 [30]	< 45° N	5000	4	20	10.9 ± 0.5	38.4 ± 1.5	17.5	15	12.4 ± 0.8	13.1 ± 1.0	0.7
Close 2013b [32]	≥45° N	5000	6	5	11.6 ± 10.0	41.3 ± 10.0	29.7	5	21.2 ± 11.6	29.6 ± 9.6	8.4
^b^Close 2013a [28]	≥45° N	5714	12	6	20.4 ± 10.4	36.5 ± 9.6	16.1	9^	20.8 ± 10.8	16.4 ± 8.8	−4.4
^b^Close 2013a [28]	≥45° N	2857	12	10	21.2 ± 10.4	34.1 ± 4.0	12.9	9^	20.8 ± 10.8	16.4 ± 8.8	− 4.4
Wyon 2016 [29]	≥45° N	18,750	1	11	13.2 ± 3.8	16.8 ± 3.2	3.6	11	16.3 ± 2.7	16.3 ± 2.6	0
Sufficient vitamin D (*N* = 57)											
Autumn (*N* = 57)											
Fairbairn 2018 [31]	≥45° S	3570	12	28	37.2 ± 7.6	44.4 ± 7.2	7.2	29	38 ± 6.8	34 ± 6.8	−4

^a^Presented in ng/mL. ^b^Close et al. compared multiple doses with one control group. ^ Indicates findings are from one study

#### Sensitivity analysis for vitamin D3 supplementation on serum 25(OH)D status

**Fig. 5:**
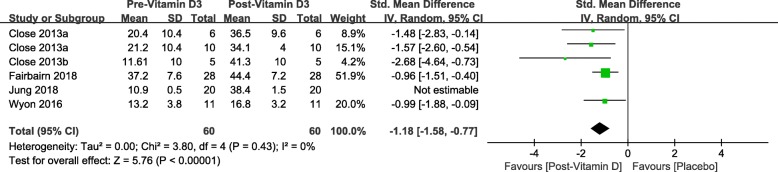
Sensitivity analysis for vitamin D3 supplementation effects on serum 25(OH)D status

Sensitivity analysis was performed by removing trials which losing > 15% participants at the end point of study from baseline assessment. Jung HC [30] demonstrated they lost 20% participants at the end point of study, therefore, Jung’s study is removed (weight = 0%) in this sensitivity analysis (Fig. 5). From this sensitivity analysis in Fig. 5, an overall beneficial effects of vitamin D3 supplementation on serum 25(OH)D still exist.

### Strength tests

**Fig. 6:**
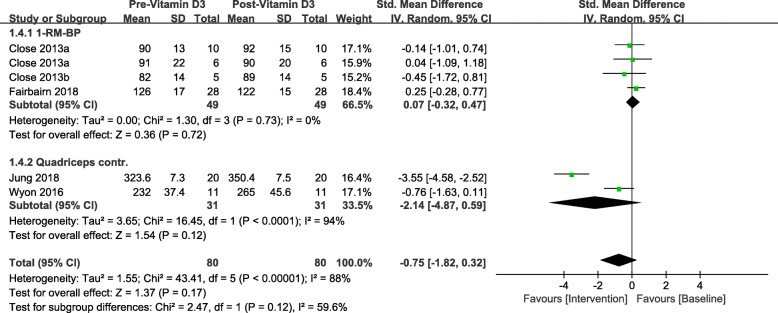
Forest plot for vitamin D3 supplementation effects on muscle strength

Total sample size in this study is 149 including both intervention and placebo. Table 5 shows the strength changes between pre and post- vitamin D3 intervention for one repetition maximum Bench Press (1-RM BP) and maximal quadriceps contraction. After generating the forest plot for different strength tests subgroups in Fig. 6, we found neither 1-RM BP (SMD 0.07, 95% CI: − 0.32 to 0.47, *P* = 0.72) nor maximal quadriceps contraction (SMD -2.14, 95% CI: − 4.87 to 0.59, *P* = 0.12) significantly improved based on current evidence. And, furthermore, no overall effect on muscle strength was observed based on included RCTs (SMD -0.75, 95% CI: − 1.82 to 0.32, *P* = 0.17).

**Table 5:** Strength outcome measures

Reference	Vitamin D Daily Dosage	*N* = 149	1-RM BP (kg)			Quadriceps Contr. (N·m)		
	IU		Pre	Post	Change	Pre	Post	Change
^a^Jung 2018 [30]	5000	20				323.6 ± 7.3	350.4 ± 7.5	25.8
	0	15				329.9 ± 8.4	339.2 ± 8.7	9.3
Fairbairn 2018 [31]	3570	28	126 ± 17	122 ± 15	−4			
	0	29	122 ± 17	123 ± 16	1			
^b^Close 2013b [32]	5000	5	82 ± 14	88.5 ± 14	6.5			
	0	5	82 ± 14	84.5 ± 14	2.5			
Close 2013a [28]	5714	6	91 ± 22	90 ± 20	−1			
	2857	10	90 ± 13	92 ± 15	2			
	0	9	79 ± 17	79 ± 18	0			
^c^Wyon 2016 [29]	18,750	11				232 ± 37.4	265 ± 45.6	33
	0	11				239 ± 65.9	239 ± 63.7	0

^a^Did 3 maximal quadriceps contraction at 60°/s; ^b^Baseline measured from an average from 10 participants; ^c^Did 3 maximal quadriceps contraction at 30°/s

## Discussion

### Summary of Main findings

#### Serum 25(OH)D concentrations

Our updated meta-analysis findings suggest that the supplementation of vitamin D3 over 4 to 12 weeks with a daily dosage over 2857 IU in winter can be of help to bring athletes’ serum 25(OH)D concentrations from insufficient to sufficient. From Figs. 4 and 5, we can make the generalization that there is enhancement effect on standard mean serum 25(OH)D concentrations from vitamin D3 supplementation. 4-weeks supplementation of 5000 IU vitamin D3 supplementation brought participants serum 25(OH)D from deficiency to sufficiency at 33.3° N latitudes in winter [30]. The sensitivity analysis also observed an overall beneficial effects of vitamin D3 supplementation on serum 25(OH)D.

#### Muscle strength

In order to generate consistent result for pooled mean difference between post-intervention results and baseline profile, each subgroup included two to three trials that contributed to the pooling of standard mean difference of the strength measurement. 1-RM BP [28, 31, 32] was not improved significantly (SMD 0.07, 95% CI: − 0.32 to 0.47, *P* = 0.72). Neither Wyon et al. [29] nor Jung [30] observed significant increase in maximal quadriceps contraction, and the overall effect of maximal quadriceps contraction was not significant (SMD -2.14, 95% CI: − 4.87 to 0.59, *P* = 0.12). Furthermore, there was no significant overall effect of vitamin D3 intervention on muscle strength in this meta-analysis (SMD -0.75, 95% CI: − 1.82 to 0.32, *P* = 0.17) shown in Fig. 6.

### Overview of overall quality

PRISMA criteria of Cochrane reviews were used to ensure quality and rigorous methodology. The selection and review process was independently conducted by two reviewers. Our conclusions are made based on findings from up-to-date officially published RCTs to ensure the quality of this systematic reviews and meta-analyses.

### Strengths and limitations

With limited RCTs available after screening and selection assessment process for this meta-analysis, this study only has data from five RCTs of certain variables inherent to meta-analysis including different supplementation dosage, outcome measurements, sports and training routines, which may introduce confounders with limited subjects.

Our study has certain limitations inherent to systematic reviews and meta-analysis and cannot be disregarded, such as year-round indoors training (like Judo and Taekwondo) may significantly reduce serum 25(OH)D compared with outdoors sports. Furthermore, muscle strength can be more important in certain sports since taekwondo and judo athletes pay more attention to enhancing strength then soccer players. With limited RCTs observed vitamin D3 supplementations on muscle strength, it is therefore not feasible to adjust for different variables, like measurement performed during different season of the year, sport professions, sunlight exposure, specific age groups, genders, type of diets (such as Mediterranean diet, vegan diet, Ketogenic diet), etc.

The finding of having no overall effect of vitamin D3 on muscle strength in this study could due to small sample size and not being able to stratify included athletes for better control when pooling and summarizing each outcome. The level of current evidence of this meta-analysis is estimated as moderate to high for elevating serum 25(OH)D concentrations with appropriate dosage and duration, but, low evidence for enhancing muscle strength have been observed.

The sample sizes in these included trials were small [28–32], varying from 10 to 57, and between-study baseline serum 25(OH)D status heterogeneity was large. The populations that been studied were very diverse with different sport professions, nationalities, living latitudes, and there were only 5 athletes received vitamin D3 intervention in a study [31]. Sport activities that athletes undertook were also varied within one study which included mixed athletes from both football and rugby [28]. Sunlight exposure is considered to be crucial for human body vitamin D synthesis under the skin [33], and athletes [34] with weight management protocols and limited sunlight ultraviolet exposure, for example, figure skating athletes [35] and ice hockey players [36–38], were reported to have high prevalence of vitamin D deficiency.

In the process of RCTs selection from fully assessed articles, nine studies [39–47] reported vitamin D supplementation had beneficial effect on elevating serum 25(OH)D, but not establishing any association between vitamin D3 supplementation and muscle strength. There are three studies [43–45] indicating that vitamin D2 supplementation significantly increased serum 25(OH)D2 concentration, but decreased serum 25(OH)D3 concentration and had no overall effects on strength tests. In mice models restricted to either vitamin D2 alone or vitamin D3 alone in its diet, vitamin D2 fed mice had superior bone health regarding bio-markers compared with vitamin D3 fed mice by the week 16 [48]. In contrast, vitamin D2 supplementation was less effective than vitamin D3 in maintaining healthy serum 25(OH)D status reported by other researchers [49–53]. The Longitudinal Aging Study Amsterdam [54] indicates that down-regulated vitamin D and elevated parathyroid hormone levels can indicate loss of muscle strength.

Though, vitamin D3 supplementation was reported to improve physical fitness [54–57], high-quality RCTs of vitamin D3 supplementation for athletes are still badly in need.

### Implications for research and practice

This meta-analysis looked at up-to-date vitamin D3 supplementation effect on serum 25(OH)D and muscle strength from RCTs. After quantitative synthesis in this systematic review and meta-analysis, we have clearly showed that there was a small effect size and little evidence for improved muscle strength with vitamin D3 intervention. Therefore, even though we observed improved 25(OH)D in this meta-analysis, we cannot make the conclusion that vitamin D3 supplementation have beneficial effect on muscle strength.

## Conclusions

Though, sufficiency achieved in serum 25(OH)D concentrations with a dosage of 2850–5000 IU vitamin D3 for over 4 weeks [27, 28, 32] were observed in RCTs, the overall effect of vitamin D3 administration on muscle strength was not significant. For the future, well-designed RCTs are still needed to look at the impact of vitamin D3 supplementation among different athletes in the aspect of muscle strength and athletic performance.

## Data Availability

All data and materials have open access to the public upon reasonable requests.
